# National Economic Development Status May Affect the Association between Central Adiposity and Cognition in Older Adults

**DOI:** 10.1371/journal.pone.0148406

**Published:** 2016-02-10

**Authors:** Asri Maharani, Gindo Tampubolon

**Affiliations:** 1 Medical Faculty, Brawijaya University, Malang, Indonesia; 2 Cathie Marsh Institute for Social Research, University of Manchester, Humanities Bridgeford Street Building, Oxford Road, Manchester, M13 9PL, United Kingdom; Nathan Kline Institute and New York University School of Medicine, UNITED STATES

## Abstract

**Background:**

Obesity is becoming a global problem, rather than one found only in developed countries. Although recent studies have suggested a detrimental effect of obesity on cognition, studies of the relationship between obesity and cognition among older adults have been limited to developed countries. We aimed to examine the associations between central obesity, as measured by waist circumference, and cognition level in adults aged 50 years and older in England and Indonesia.

**Methods:**

We used linear regression models to analyse these associations and multiple imputation to manage missing data. The 2006 English Longitudinal Study of Ageing Wave 3 is the source of data from England, while data from Indonesia is sourced from the 2007 Indonesian Family Life Survey Wave 4.

**Findings:**

Centrally obese respondents had lower cognition levels than non-centrally obese respondents in England. In contrast, central adiposity had a statistically significant positive association with cognition in Indonesia. Higher levels of education and higher economic status were associated with higher cognitive ability, while age was associated with lower cognition in both countries. Elevated C-reactive protein (CRP) concentrations and smoking behaviour, both linked to higher risk of obesity, were negatively associated with cognitive ability among older adults in England, but they had no statistically significant association with cognition among Indonesians.

**Interpretation:**

The contradictory findings on obesity and cognition in England and Indonesia not only create a puzzle, but they may also have different policy implications in these countries. Reducing the prevalence of obesity may be the main focus in England and other developed countries to maintain older adults’ cognition. However, Indonesia and other developing countries should place more emphasis on education, in addition to continued efforts to tackle the double burden of malnutrition, in order to prevent cognitive impairment among older adults.

## Introduction

Over the past three decades obesity has become a global public health problem as the number of overweight and obese people increased more than twofold from 857 million in 1998 to 2.1 billion in 2013 [1]. Ominously, two in three obese people live in developing countries. This high burden of obesity coupled with the still significant prevalence of under-nutrition has created a double burden of malnutrition in these countries. The double burden of malnutrition is the persistence of diseases resulting from both under-nutrition along with over-nutrition within a population [2,3]. Obesity is well recognised as having associations with increased risk of cardiovascular disease, type 2 diabetes mellitus and cancer [4,5]. Recently, scholars have given more attention to the associations between obesity and cognitive function, independent of the effect of medical comorbidities [6,7]. Elias and colleagues reported that obesity had a significant and negative association with cognitive function among male participants, independent of the effect of diabetes mellitus in the U.S [8]. Cournot and colleagues later found that higher body mass index (BMI), a measure of obesity, was associated with lower cognitive function after adjustment for demographic and characteristics comorbidities in France [9]. Supporting those studies, Fergenbaum and colleagues found that obese participants in Canada had a four times the risk of lower cognitive function than their non-obese peers [10].

However, these studies all took place in developed countries, leaving evidence of the associations between obesity and cognition in developing countries undiscovered; this is a serious omission given the number of obese people in these countries. This study thus aimed to compare the consequences of obesity on cognition among older adults in the contexts of developed and developing countries using data from England and Indonesia. England has the highest prevalence of overweight and obesity in Europe with more than half of its population aged 20 and older (66% of men and 57% of women) being overweight or obese in 2013 [1,11]. Indonesia is an interesting case of a developing country with a considerable obesity problem as the nutritional profile of its large population is undergoing major shifts [12]. Despite the persistent high burden of under-nutrition in Indonesia, the data from national basic health research show that the prevalence of overweight and obesity among adults (aged 18 and over) increased markedly from 19% in 2007 to almost 29% in 2013, which is equivalent to about 72 million people: more than the total population of England [13].

Our study focused on older adults (50 years and older) as they are at a higher risk of cognitive decline than any other age group [14]. Declining cognition is one of the main causes of loss of independence in this age group [15]. However, measuring the relationship between obesity and cognition among older adults is more complex than it is with other age groups. Some studies have reported that general obesity (measured as BMI) in later life is associated with lower cognitive function [8,9,10], while others have reported the opposite [16,17]. Smith and colleagues suggested that BMI is not a good measure of body composition for older adults [6]. Testosterone retained in the body fat of male respondents and higher levels of leptin in respondents may protect their cognitive function. Our research extends previous studies by using central adiposity, measured by waist circumference, as a predictor of cognitive function. Waist circumference is known to be more accurate than BMI for the purposes of identifying risk for dementia and obesity comorbidities such as cardiovascular diseases [18,19]. Jagus and colleagues found central adiposity to be associated with decreased hippocampal volumes and increased white matter hyperintensities, which may affect the structure of the brain and bring about cognitive decline [20].

This study further included C-reactive protein (CRP) in the analysis. Studies using samples of older people in developed and developing countries repeatedly show the negative association between CRP and cognitive function [21–24]. For example, Gunstad et al. found that CRP was associated with lower global cognitive performance, memory and visuospatial abilities among 126 older cardiovascular patients [22]. More recent study by Bettcher et al. supported those findings and found higher CRP was related to lower performance on delayed recall task [23]. However, none of those studies implicate CRP in the relationship of obesity and cognitive function.

Our study compared the relationships between central adiposity and cognitive function in England and Indonesia, taking into account demographic factors, health behaviour and the presence of comorbidities through the use of comparable surveys from these countries. For comparison, this study provides an analysis using BMI.

## Materials and Methods

### Data sources

We used two nationally representative surveys to compare the consequences of central adiposity on cognitive abilities in England and Indonesia: the 2006 English Longitudinal Study of Ageing (ELSA) Wave 3 and the 2007 Indonesian Family Life Survey (IFLS) Wave 4 [25–26]. We chose those waves of the surveys as they had similar time frames and included the same measure of cognitive function. ELSA Wave 3 collected information on the personal, socio-economic and health circumstances of non-institutionalised adults aged 50 years and older in England, while IFLS Wave 4 provided information on similar topics among non-institutionalised adults aged 15 years and older in Indonesia. We included respondents aged 50 years and older who had never experienced a stroke event prior to the cognitive testing. Our final sample comprised 8,189 individuals in England and 2,594 individuals in Indonesia.

We used the available measure of cognitive ability operationalised comparably across both surveys, namely, episodic memory. In both surveys, episodic memory was measured by immediate and delayed word recall. Immediate recall of ten words was used as a measure of short-term memory, while delayed recall of the same words (i.e., 20 minutes after initial word presentation) was used as a measure of long-term memory. Episodic memory is one of the more sensitive cognitive domains for older adults as it is among the first cognitive abilities to decline with age [27]. We use the raw data on the number of correct responses of immediate (10 words) and delayed word recall (10 words) from a total possible points of 20 to measure episodic memory of the respondents in our analyses [28,29]. We treated the data as continuous.

We defined central adiposity in terms of large waist circumference—larger than 90 cm in men and larger than 80 cm in women [30]. We divided body weight into four categories defined as ‘obese’ (BMI ≥ 25 kg/m^2^), ‘overweight’ (BMI 23–25 kg/m^2^), ‘normal’ (BMI 18.5–23 kg/m^2^) and ‘underweight’ (BMI < 18.5 kg/m^2^) [30]. We included demographic and socio-economic information as well as health behaviour and the presence of associated medical conditions as the covariates of cognition. We categorised the levels of education completed by respondents into primary school, secondary school and college and higher, while marital status was classified as single, married, divorced or widowed. We used different measures of economic status: the aggregate of private pension wealth and state pension wealth in England and expenditure per capita in Indonesia. We used tertiles of economic status, using the poorest tertile as the reference. We included smoking behaviour as an indicator of health risk and categorised respondents as either current smokers or non-current smokers. We classified a respondent as hypertensive if he/she had persistent elevation of blood pressure (≥ 140/90 mm Hg) or was taking antihypertensive medications [31]. The indicator of dyslipidaemia in this study was the ratio of total cholesterol and high-density lipoprotein cholesterol. The cut-off point was defined as a total cholesterol/HDL cholesterol ratio of ≥ 5 [32]. C-reactive protein (CRP) was used to measure general levels of inflammation, whose negative association with cognition has been reported in previous studies [33,34]. We entered the CRP variable as a log-transformed continuous variable to make the distribution more symmetric and to reduce the effect of outliers.

### Statistical analysis

We used simple linear regression analysis to test the bivariate associations. We then performed multiple linear regression analysis to quantify the associations between the level of cognition (as measured by episodic memory) and central adiposity in the first model, independent of several potentially confounding factors (age, gender, education, marital status, economic status, the presence of hypertension, dyslipidaemia, CRP level and smoking status) [8,34–36]. We employed linear regression with each test score as dependent variable and those potential confounding factors as the predictor variables to adjust the effect of the covariates. We also examined the association between the level of cognition and total obesity and included the confounding factors mentioned above in the second model. All analyses of the data on England and Indonesia were conducted separately.

Data were missing from both studies; this can reduce the precision of estimates. To avoid the potential bias that could have arisen through mishandling of incomplete data, we applied multiple imputation [37]. We used all predictors together to replace each missing value with a set of plausible values that represent the uncertainty regarding the right value to impute. We performed sensitivity analysis by analysing the multiple imputed data and the original data and comparing the results (see S1 and S2 Tables). All statistical analyses were conducted using Stata 12.0.

## Results

A total of 8,189 participants in England and 2,594 participants in Indonesia aged 50 years and over were initially included in this study. The descriptive statistics (see Table 1) show that older adults from Indonesia performed less well than those from England on the test of cognitive ability as they were able to recall four words fewer than their British peers. The proportion of participants with central adiposity in England (84%) was more than twice the proportion in Indonesia (36%). Results were similar when obesity was measured using BMI. The proportion of obese British respondents (31%) was five times that of Indonesian respondents (6%). Underweight was still a significant problem in Indonesia as more than 17% of participants in this country had a BMI of less than 18.5 kg/m^2^. The British participants were on average five years older than the Indonesian participants, reflecting the higher life expectancy in developed countries. The proportion of male respondents in England and Indonesia were similar, accounting respectively for 45% and 47% of respondents.

**Table 1:** Sample characteristics and exploratory bivariate analysis.

	England (N = 8,189)			Indonesia (N = 2,594)		
	Mean ± SD (%)	Association with cognitive function*	Missing data (%)	Mean ± SD (%)	Association with cognitive function*	Missing data (%)
Cognitive function	10.53 ± 3.56		0.15	6.84 ± 3.15		17.73
Centrally obese	84.35	-0.69 (0.11) ‡	2.52	36.45	0.59 (0.14) ‡	0.27
**BMI**			4.14			1.35
Underweight	0.84	-0.46 (0.44) ‡		17.66	-1.17 (0.19) ‡	
Normal	26.04	0.00		57.56	0.00	
Overweight	41.91	-0.38 (0.10) ‡		19.30	0.78 (0.17) ‡	
Obese	31.21	-0.51 (0.10) ‡		5.47	1.03 (0.28) ‡	
Age	65.81 ± 9.7	-0.15 (0.00) ‡	0	60.59 ± 8.70	-0.10 (0.01) ‡	0
Male	45.10	-0.61 (0.08) ‡	0	47.88	0.53 (0.14) ‡	0
**Education:**			0.24			21.36
Primary school or less	40.89	0.00		66.42	0.00	
Secondary school	27.15	1.84 (0.09) ‡		27.16	1.88 (0.15) ‡	
College or higher	31.96	2.36 (0.09) ‡		6.42	3.04 (0.09) ‡	
**Marital status:**			0			0
Single	5.79	-0.51 (0.17) ‡		0.93	0.60 (0.67)	
Married	67.46	0.00		75.71	0.00	
Divorce	11.71	0.07 (0.12)		3.08	-0.85 (0.40) †	
Widowed	15.04	-1.84 (0.11) ‡		20.28	-0.98 (0.18) ‡	
Having hypertension	51.19	-0.97 (0.08) ‡	2.43	44.80	-0.04 (0.14)	0.73
Having dyslipidaemia	12.48	-0.15 (0.13)	24.67	46.27	0.44 (0.14) ‡	1.85
CRP (mg/dL)	2.41 ± 2.13	-0.37 (0.05) ‡	29.77	1.67 ± 1.72	0.11 (0.10)	34.50
Current smoker	14.71	-0.05 (0.13) ‡	28.47	39.94	0.18 (0.14)	0

Note: * Reported are coefficients (standard errors). Sig.: †: significant at 5% or less; ‡: significant at 1% or less.

The differences in educational attainment between people in developed and developing countries are well reflected in our results. The present-day older adults from England were six-fold more likely than those from Indonesia to have earned a college degree or attained a higher level of education (master and doctoral degrees). Most participants in both countries were married (67% in England and 75% in Indonesia). The percentage of British older adults with hypertension was slightly higher than that of Indonesian older adults (51% versus 44%). Despite the low proportion of obese Indonesians in this study, their likelihood of having dyslipidaemia (46%) was four times higher than that of the obese British respondents (12%). The mean CRP among British participants was higher than that of Indonesian participants at 2.41 mg/dL and 1.67 mg/dL, respectively. Smoking was more common among Indonesian respondents (39%) than British respondents (14%).

**Fig 1:**
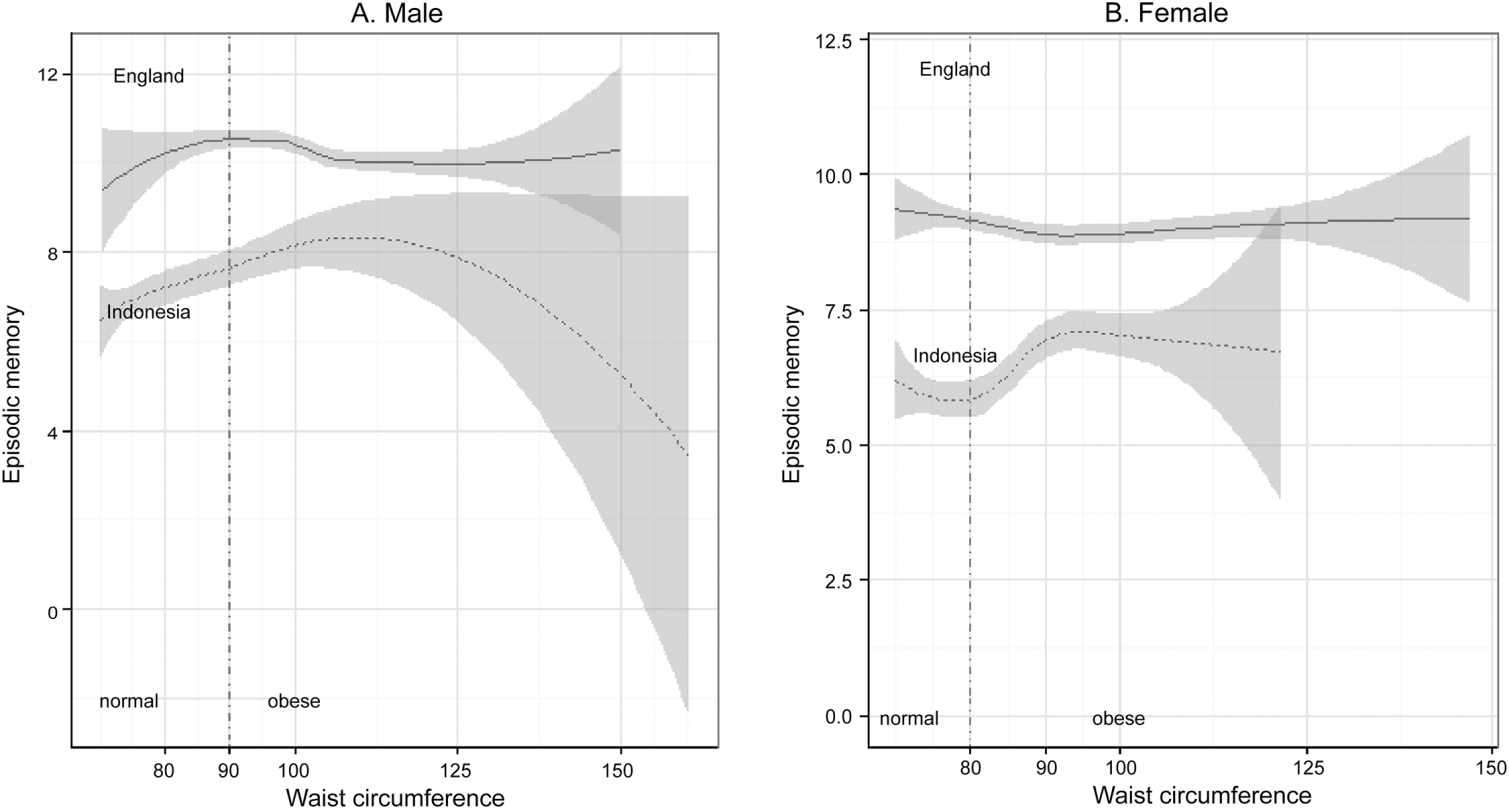
Episodic memory level and waist circumference between England and Indonesia.

**Fig 2:**
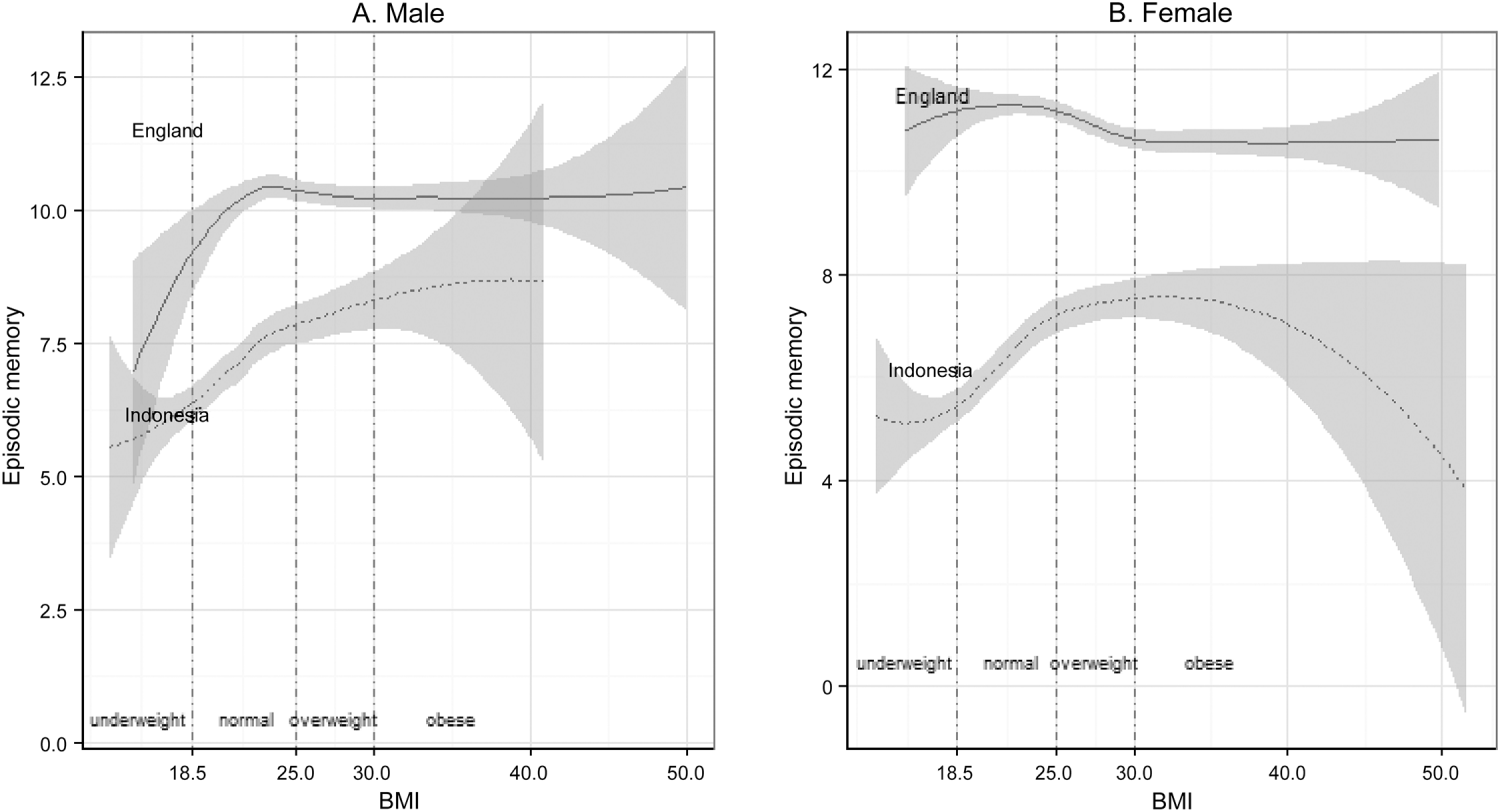
Episodic memory level and BMI between England and Indonesia.

Figs 1 and 2 plotted the local regressions of cognitive level as a function of obesity among males and females, with separate curves for England and Indonesia using Model 1. Fig 1A and 1B shows that among non-centrally obese respondents, higher waist circumference was associated with better cognitive ability in both countries. However, its associations with centrally obese respondents were different in the two countries. Larger waist circumference was negatively associated with the cognitive ability of centrally obese males in England. In Indonesia, larger waist circumference was related to higher cognition among centrally obese males up to 110 cm, but lower cognition beyond that point. The wide confidence intervals on this association may be due to the small number of males in the sample with very large waist circumferences. Similarly, waist circumference had a negative association with the cognitive ability of centrally obese females in England with waist circumferences up to 93 cm but had a positive association in Indonesia. Fig 2A and 2B show that the associations between BMI and the cognitive function of obese respondents were different in the two countries. Higher BMI was associated with lower cognitive function in both overweight and obese respondents of both genders in England, but the same association was found only among males in Indonesia.

The multiple linear regression results of imputed data for the association of central obesity and cognitive function (adjusted for comorbidities, health behaviour and socio-demographic characteristics) in England and Indonesia using unimputed data are shown in Table 2. It displays the unstandardised regression coefficient (B), the standard error of the regression coefficients (SE B) and the standardised regression coefficients (β). Central adiposity had different relationships with cognition among respondents in the two countries. Model 1 shows that centrally obese participants in England performed less well on cognition tests than their non-centrally obese peers (β = -0.07, p<0.01), while the cognitive function of centrally obese participants in Indonesia was higher than that of their non-centrally obese peers (β = 0.09, p<0.01). When the models were adjusted for comorbidities and smoking behaviour, central obesity still significantly correlated with lower cognition in England (β = -0.05, p<0.01) and higher cognition in Indonesia (β = 0.10, p<0.01). The negative association between central obesity and cognition in England (β = -0.03, p<0.05) remained in the final model (Model 3), where the potential confounders and socio-demographic characteristics were included. The positive association between central obesity and cognition in Indonesia vanished in the third model in the analysis using unimputed data. However, the analysis using imputed data shows that the positive association between central obesity and cognition is still significant with p<0.05 (S1 Table).

**Table 2:** Multiple linear regression results for the association between waist circumference and cognitive function using non-imputed data. Note: B (SE B): Reported are coefficients (standard errors). β: Reported are standardised beta coefficients. Sig.: †: significant at 5% or less; ‡: significant at 1% or less.

	England						Indonesia					
	Model 1		Model 2		Model 3		Model 1		Model 2		Model 3	
	*B* (SE *B*)	β	*B* (SE *B*)	β	*B* (SE *B*)	β	*B* (SE *B*)	β	*B* (SE *B*)	β	*B* (SE *B*)	β
Centrally obese	-0.69(0.10)‡	-0.07	-0.54(0.15)‡	-0.05	-0.31(0.14)†	-0.03	0.59(0.14)‡	0.09	0.69(0.19)‡	0.10	0.28(0.21)	0.04
Having hypertension			-0.74(0.11)‡	-0.10	-0.06(0.10)	-0.00			0.03(0.17)	0.00	0.27(0.18)	0.04
Having dyslipidaemia			0.13(0.17)	0.01	0.01(0.15)	0.00			0.30(0.17)	0.04	-0.19(0.17)	-0.03
Log CRP			-0.28(0.06)‡	-0.07	-0.13(0.05)†	-0.03			0.06(0.10)	0.01	0.12(0.10)	0.03
Current smoker			-0.09(0.16)	-0.00	-0.40(0.15)†	-0.03			0.41(0.19)†	0.06	-0.14(0.26)	-0.02
Age					-0.14(0.00)‡	-0.37					-0.09(0.01)‡	-0.22
Male					-1.21(0.11)‡	-0.17					0.56(0.27)†	0.08
**Education, ref: Primary school or less**												
Secondary school					1.06(0.12)‡	0.13					1.47(0.20)‡	0.21
College or higher					1.54(0.12)‡	0.20					2.36(0.35)‡	0.19
**Marital status, ref: Married**												
Single					-0.88(0.23)‡	-0.05					0.73(0.93)	0.02
Divorce					-0.15(0.16)	-0.01					-0.46(0.56)	-0.02
Widowed					-0.13(0.15)	-0.01					0.10(0.26)	0.01
**Economic status, ref: 1st tertile**												
2nd tertile					0.14(0.12)	0.01					0.05(0.23)	0.00
3rd tertile					0.85(0.14)‡	0.11					0.40(0.24)	0.06
Constant	11.16(0.09)‡		11.66(0.14)‡		20.51(0.48)‡		6.63(0.09)‡		6.37(0.19)‡		11.66(0.74)‡	
N	7971		3931		3623		2130		1361		1144	

Table 3 presents the results for the association of general obesity (as measured by BMI) and cognitive function among older adults in England and Indonesia. As with earlier models, obesity showed different associations with cognition in those countries. In England, the overweight (β = -0.05, p<0.01) and obese respondents (β = -0.06, p<0.01) had lower cognitive function than respondents with normal BMIs. However, in Indonesia, the overweight (β = 0.09, p<0.01) and obese respondents (β = 0.07, p<0.01) performed better on cognition tests than those with normal BMIs. The association between overweight and cognitive function in Indonesia, however, vanished after the model took into account the potential confounders and socio-demographic characteristics (Model 3). In England, overweight (β = -0.04, p<0.05) and obesity (β = -0.05, p<0.01) were still significantly associated with lower cognitive ability in the same model.

**Table 3:** Multiple linear regression results for the association between BMI and cognitive function using non-imputed data.

	England						Indonesia					
	Model 1		Model 2		Model 3		Model 1		Model 2		Model 3	
	*B* (SE *B*)	β	*B* (SE *B*)	β	*B* (SE *B*)	β	*B* (SE *B*)	β	*B* (SE *B*)	β	*B* (SE *B*)	β
**BMI, ref: Normal**												
Underweight	-0.46(0.44)	-0.01	-0.17(0.64)	-0.00	0.66(0.62)	0.01	-1.17(0.19)‡	-0.13	-1.50(0.26)‡	-0.15	-1.00(0.28)‡	-0.10
Overweight	-0.38(0.09)‡	-0.05	-0.23(0.13)	-0.03	-0.29(0.12)†	-0.04	0.77(0.17)‡	0.09	0.85(0.20)‡	0.11	0.27(0.21)	0.03
Obese	-0.51(0.10)‡	-0.06	0.07(0.16)	-0.00	-0.43(0.14)‡	-0.05	1.02(0.28)‡	0.07	1.26(0.33)‡	0.10	0.49(0.33)	0.04
Having hypertension			-0.76(0.11)‡	-0.10	-0.04(0.10)	-0.00			-0.08(0.17)	-0.01	0.20(0.18)	0.03
Having dyslipidaemia			0.06(0.17)	0.00	0.04(0.15)	0.00			0.15(0.17)	0.02	-0.26(0.17)	-0.04
Log CRP			-0.31(0.06)‡	-0.08	-0.09(0.05)	-0.02			0.06(0.09)	0.01	0.10(0.10)	0.02
Current smoker			-0.11(0.16)	-0.01	-0.46(0.15)‡	-0.04			0.55(0.18)‡	0.08	0.00(0.26)	0.00
Age					-0.14(0.00)‡	-0.37					-0.08(0.01)‡	-0.20
Male					-1.19(0.11)‡	-0.16					0.50(0.27)	0.08
**Education, ref: Primary school or less**												
Secondary school					1.03(0.12)‡	0.13					1.45(0.20)‡	0.21
College or higher					1.54(0.12)‡	0.20					2.27(0.35)‡	0.19
**Marital status, ref: Married**												
Single					-0.92(0.24)‡	-0.05					0.88(0.92)	0.02
Divorce					-0.16(0.16)	-0.01					-0.27(0.55)	-0.01
Widowed					-0.14(0.15)	-0.01					0.18(0.26)	0.02
**Economic status, ref: 1st tertile**												
2nd tertile					0.12(0.13)	0.01					-0.02(0.23)	-0.00
3rd tertile					0.83(0.14)‡	0.11					0.27(0.24)	0.04
Constant	10.94(0.07)‡		11.40(0.12)‡		20.53(0.49)‡		6.80(0.08)‡		6.62(0.18)‡		11.33(0.73)‡	
N	7971		3931		3623		2130		1361		1144	

Note: B (SE B): Reported are coefficients (standard errors). β: Reported are standardised beta coefficients. Sig.: †: significant at 5% or less; ‡: significant at 1% or less.

The results of this study clearly illustrate the negative association between underweight and cognitive function, especially in developing countries. Our study shows that the underweight respondents in Indonesia had lower cognitive ability than those of normal weight (β = -0.13, p<0.01). The association remained significant when the model was adjusted to account for comorbidities, smoking behaviour, and socio-demographic factors (β = -0.10, p<0.01). Despite their lack of significance, the results in England also show trend toward a negative association between underweight and cognition level. Table 4 shows the small effect sizes for obesity and cognition in all models.

**Table 4:** The effect sizes for cognitive function using non-imputed data.

Regression model	England		Indonesia	
	Partial R^2^	Descriptor	Partial R^2^	Descriptor
***Centrally obese***				
Model 1	0.005	Small	0.008	Small
Model 2	0.003	Small	0.009	Small
Model 3	0.001	Small	0.001	Small
***BMI*: *ref*: *Normal***				
*Underweight*				
Model 1	0.000	Small	0.017	Small
Model 2	0.000	Small	0.022	Small
Model 3	0.000	Small	0.011	Small
*Overweight*				
Model 1	0.001	Small	0.009	Small
Model 2	0.000	Small	0.012	Small
Model 3	0.001	Small	0.001	Small
*Obese*				
Model 1	0.003	Small	0.006	Small
Model 2	0.000	Small	0.010	Small
Model 3	0.002	Small	0.001	Small

Several potential confounders and socio-demographic characteristics showed significant associations with cognitive function among older adults in England and Indonesia. Higher educational attainment and better economic status were correlated with higher cognition in all models in both countries. Age had a negative and significant correlation with cognition in all models in both countries. Males had lower cognitive function than females in England, but higher cognition in Indonesia. Single participants had lower cognitive function than married participants in England. Log CRP and current smoker status both had negative effects on cognitive function in England. To ascertain robustness we repeated the analysis using only complete data (S1 and S2 Tables). Compared to the analysis using multiple imputed data, the analysis using incomplete data gave less precise standard errors. The absence of substantial differences between them indicated that the results were robust.

## Discussion

Although the prevalence of obesity has increased sharply in developing countries [1], there is a paucity of investigation revealing its association with cognition in these countries. This study, therefore, investigates this association using a recent national sample from Indonesia. Further, it provides a comparative analysis using a comparable national sample from England. The results are puzzling as central obesity showed different associations with cognition in England and Indonesia. Following previous research in developed countries, the present study, using waist circumference as the measure, provides an additional source of empirical support for the generally established view that obesity has a significant association with lower cognition [8–10].

The centrally obese older adults in England had lower levels of cognition than their non-centrally obese peers, although the effect size was small. In contrast, our results in Indonesia show that the centrally obese older adults had higher cognition levels than their non-centrally obese peers with similar effect size. Since this study is among the first to examine the consequences of obesity on cognition in the context of a developing country, the association between obesity and higher cognition level should be interpreted with caution. Education and economic status are among the important confounders of cognition that could mask the true relationship between obesity and cognitive function. We explored the potential of that explanation by including education and economic status as covariates in our analysis. However, the positive association between obesity and cognition remains significant, even after including education and economic status.

One explanation for this puzzle could involve reverse causality (cognitive function as a predictor of obesity). Longitudinal studies in developed countries have found that lower cognitive function leads to higher BMI by means of several mechanisms [38–40]. The first of these is that cognitive function encompasses a number of neuropsychological concepts, including inhibitory control, attention control and problem solving, which influence the ability to control eating urges [38]. The second mechanism is that individuals with higher executive cognitive function have a greater ability to formulate long-term health plans that include eating healthy foods. They may be more likely to implement such plans [40].

Yet the results in Indonesia suggest that the mechanisms showing a positive correlation between cognition dysfunction and obesity do not apply in developing countries. The reverse causality in those countries may be explained by the fact that people with higher cognitive level tend to engage in sedentary work which require less energy expenditure of physical energy [41]. Not only do sedentary workers engage in less physical activity, but they also tend to eat commercially prepared foods that often lead to increased body weight [42]. The positive association between cognitive ability and obesity in developing countries indicates that those lifestyles are hardly changed in later life, although people may have more time to exercise. Unfortunately, the cross-sectional design of our research does not allow us to test these explanations. To better comprehend the mechanism underlying these puzzling results, prospective studies starting in youth or middle age and involving a long period of follow-up will be required.

Other than its cross-sectional design, our study is limited by the availability of comparable data on cognition in Indonesia and England. Previous studies on obesity and cognition used various tests of cognition [8–10]. For example, Cournot et al. used the Wechsler Adult Intelligence Survey (WAIS) Digit–Symbol Substitution Subtest, which is highly sensitive to the effects of ageing [9]. The data used in this study was not originally designed to compare cognition between countries; hence the comparable information is only the episodic memory. Research using additional cognitive tests will provide a better understanding of the relationship between obesity and cognitive function in both developed and developing countries.

The differing associations between obesity and cognitive function in England and Indonesia strengthen the view that results acquired in the developed world may not be straightforwardly applied to the developing world. These facts should perhaps lead to—different policy priorities in England and Indonesia. Given the significant negative association between obesity and cognitive function in England, this and other developed countries may wish to put considerable effort into reducing the prevalence of obesity in order to maintain the cognitive function of their older populations. Such efforts are also recommended to prevent cognitive impairment in Indonesia and other developing countries. However, these countries should put more effort into education: our findings suggest that interventions to enhance educational opportunities throughout life could potentially prevent cognitive function decline, thereby substantially improving quality of life among older adults in Indonesia.

## Supporting Information

S1 Table Multiple linear regression results for the association between waist circumference and cognitive function using imputed data. (DOCX)

S2 Table Multiple linear regression results for the association between BMI and cognitive function using imputed data. (DOCX)

S1 Fig Distribution of episodic memory level in England and Indonesia. (TIF)
