# Supplementary material for: National Economic Development Status May Affect the Association between Central Adiposity and Cognition in Older Adults
Source: PLoS One. 2016 Feb 10;11(2):e0148406. doi: 10.1371/journal.pone.0148406 (PMC4749166; doi:10.1371/journal.pone.0148406)
Supplement: S1 Table — (DOCX) [file pone.0148406.s001.docx]

**S1 Table:** Multiple linear regression results for the association between waist circumference and cognitive function using imputed data

|  | **England (N=8189)** | | | | | | **Indonesia (N=2594)** | | | | | |
| --- | --- | --- | --- | --- | --- | --- | --- | --- | --- | --- | --- | --- |
|  | Model 1 | | Model 2 | | Model 3 | | Model 1 | | Model 2 | | Model 3 | |
|  | *B* (SE *B*) | β | *B* (SE *B*) | β | *B* (SE *B*) | β | *B* (SE *B*) | β | *B* (SE *B*) | β | *B* (SE *B*) | β |
| Centrally obese | -0.70(0.11)‡ | -0.07 | -0.36(0.12)‡ | -0.03 | -0.24(0.10)† | -0.02 | 0.61(0.14)‡ | 0.09 | 0.76(0.15)‡ | 0.11 | 0.31(0.15)† | 0.04 |
| Having hypertension |  |  | -0.86(0.08)‡ | -0.12 | -0.11(0.07) | -0.01 |  |  | -0.18(0.13) | -0.02 | 0.10(0.13) | 0.01 |
| Having dyslipidaemia |  |  | 0.02 (0.13) | 0.00 | -0.08(0.12) | -0.00 |  |  | 0.35(0.13)‡ | 0.05 | 0.04(0.12) | 0.00 |
| Log CRP |  |  | -0.26(0.06)‡ | -0.07 | -0.07(0.05) | -0.01 |  |  | 0.02(0.10) | 0.00 | 0.05(0.10) | 0.01 |
| Current smoker |  |  | -0.12(0.13) | -0.01 | -0.52(0.13)‡ | -0.05 |  |  | 0.40(0.14)‡ | 0.06 | -0.00(0.18) | -0.00 |
| Age |  |  |  |  | -0.13(0.00)‡ | -0.34 |  |  |  |  | -0.09(0.01)‡ | -0.23 |
| Male |  |  |  |  | -1.04(0.08)‡ | -0.14 |  |  |  |  | 0.50(0.19)‡ | 0.07 |
| **Education, ref: Primary school or less** |  |  |  |  |  |  |  |  |  |  |  |  |
| Secondary school |  |  |  |  | 1.11(0.09)‡ | 0.13 |  |  |  |  | 1.63(0.16)‡ | 0.22 |
| College or higher |  |  |  |  | 1.60(0.09)‡ | 0.20 |  |  |  |  | 2.47(0.30)‡ | 0.18 |
| **Marital status, ref: Married** |  |  |  |  |  |  |  |  |  |  |  |  |
| Single |  |  |  |  | -0.66(0.15)‡ | -0.04 |  |  |  |  | 0.27(0.60) | 0.00 |
| Divorce |  |  |  |  | -0.08(0.11) | -0.00 |  |  |  |  | -0.14(0.35) | -0.00 |
| Widowed |  |  |  |  | -0.21(0.11)† | -0.02 |  |  |  |  | -0.12(0.19) | -0.01 |
| **Economic status, ref: 1st tertile** |  |  |  |  |  |  |  |  |  |  |  |  |
| 2nd tertile |  |  |  |  | 0.24(0.11)† | 0.03 |  |  |  |  | 0.07(0.15) | 0.01 |
| 3rd tertile |  |  |  |  | 0.80(0.12)‡ | 0.10 |  |  |  |  | 0.53(0.16)‡ | 0.08 |
| Constant | 11.12(0.10)‡ |  | 11.42(0.11)‡ |  | 18.66(0.34)‡ |  | 6.45(0.08)‡ |  | 6.17(0.13)‡ |  | 10.74(0.47)‡ |  |

Note: B (SE B): Reported are coefficients (standard errors). β: Reported are standardised beta coefficients. Sig.: †: significant at 5% or less; ‡: significant at 1% or less.
