# Supplementary material for: National Economic Development Status May Affect the Association between Central Adiposity and Cognition in Older Adults
Source: PLoS One. 2016 Feb 10;11(2):e0148406. doi: 10.1371/journal.pone.0148406 (PMC4749166; doi:10.1371/journal.pone.0148406)
Supplement: S2 Table — (DOCX) [file pone.0148406.s002.docx]

**S2 Table:** Multiple linear regression results for the association between BMI and cognitive function using imputed data

|  | **England (N=8189)** | | | | | | **Indonesia (N=2594)** | | | | | |
| --- | --- | --- | --- | --- | --- | --- | --- | --- | --- | --- | --- | --- |
|  | Model 1 | | Model 2 | | Model 3 | | Model 1 | | Model 2 | | Model 3 | |
|  | *B* (SE *B*) | β | *B* (SE *B*) | β | *B* (SE *B*) | β | *B* (SE *B*) | β | *B* (SE *B*) | β | *B* (SE *B*) | β |
| **BMI, ref: Normal** |  |  |  |  |  |  |  |  |  |  |  |  |
| Underweight | -0.43(0.45) | -0.01 | -0.47(0.44) | -0.01 | 0.03(0.39) | 0.00 | -1.24(0.18)‡ | -0.15 | -1.23(0.18)‡ | -0.15 | -0.64(0.18)‡ | -0.77 |
| Overweight | -0.39(0.10)‡ | -0.05 | -0.15(0.11) | -0.02 | -0.15(0.09) | -0.02 | 0.83(0.18)‡ | 0.10 | 0.93(0.18)‡ | 0.11 | 0.39(0.18)† | 0.04 |
| Obese | -0.49(0.11)‡ | -0.06 | 0.03(0.12) | 0.00 | -0.31(0.11)‡ | -0.04 | 1.12(0.29)‡ | 0.08 | 1.27(0.29)‡ | 0.09 | 0.44(0.28) | 0.03 |
| Having hypertension |  |  | -0.89(0.08)‡ | -0.12 | -0.09(0.07) | -0.01 |  |  | -0.25(0.14)† | -0.04 | 0.04(0.13) | 0.00 |
| Having dyslipidaemia |  |  | 0.01(0.13) | -0.00 | -0.07(0.12) | -0.00 |  |  | 0.23(0.13) | 0.03 | 0.01(0.12) | 0.00 |
| Log CRP |  |  | -0.30(0.06)‡ | -0.07 | -0.05(0.05) | -0.01 |  |  | 0.02(0.10) | 0.00 | 0.05(0.10) | 0.01 |
| Current smoker |  |  | -0.09(0.12) | -0.00 | -0.54(0.13)‡ | -0.05 |  |  | 0.40(0.13)‡ | 0.06 | 0.05(0.18) | 0.00 |
| Age |  |  |  |  | -0.13(0.00)‡ | -0.34 |  |  |  |  | -0.08(0.01)‡ | -0.21 |
| Male |  |  |  |  | -1.04(0.08)‡ | -0.14 |  |  |  |  | 0.42(0.18)† | 0.06 |
| **Education, ref: Primary school or less** |  |  |  |  |  |  |  |  |  |  |  |  |
| Secondary school |  |  |  |  | 1.11(0.09)‡ | 0.13 |  |  |  |  | 1.60(0.16)‡ | 0.22 |
| College or higher |  |  |  |  | 1.58(0.09)‡ | 0.20 |  |  |  |  | 2.39(0.30)‡ | 0.17 |
| **Marital status, ref: Married** |  |  |  |  |  |  |  |  |  |  |  |  |
| Single |  |  |  |  | -0.67(0.15)‡ | -0.04 |  |  |  |  | 0.39(0.61) | 0.01 |
| Divorce |  |  |  |  | -0.08(0.11) | -0.00 |  |  |  |  | -0.10(0.35) | -0.00 |
| Widowed |  |  |  |  | -0.21(0.11) | -0.02 |  |  |  |  | -0.07(0.19) | -0.00 |
| **Economic status, ref: 1st tertile** |  |  |  |  |  |  |  |  |  |  |  |  |
| 2nd tertile |  |  |  |  | 0.24(0.10)† | 0.03 |  |  |  |  | 0.04(0.15) | 0.00 |
| 3rd tertile |  |  |  |  | 0.80(0.12)‡ | 0.10 |  |  |  |  | 0.48(0.16)‡ | 0.07 |
| Constant | 10.85(0.08)‡ |  | 11.22(0.09)‡ |  | 18.73(0.34)‡ |  | 6.68(0.09)‡ |  | 6.51(0.13)‡ |  | 10.51(0.47)‡ |  |

Note: B (SE B): Reported are coefficients (standard errors). β: Reported are standardised beta coefficients. Sig.: †: significant at 5% or less; ‡: significant at 1% or less.
