# Supplementary figures and images for: National Economic Development Status May Affect the Association between Central Adiposity and Cognition in Older Adults
Source: PLoS One. 2016 Feb 10;11(2):e0148406. doi: 10.1371/journal.pone.0148406 (PMC4749166; doi:10.1371/journal.pone.0148406)

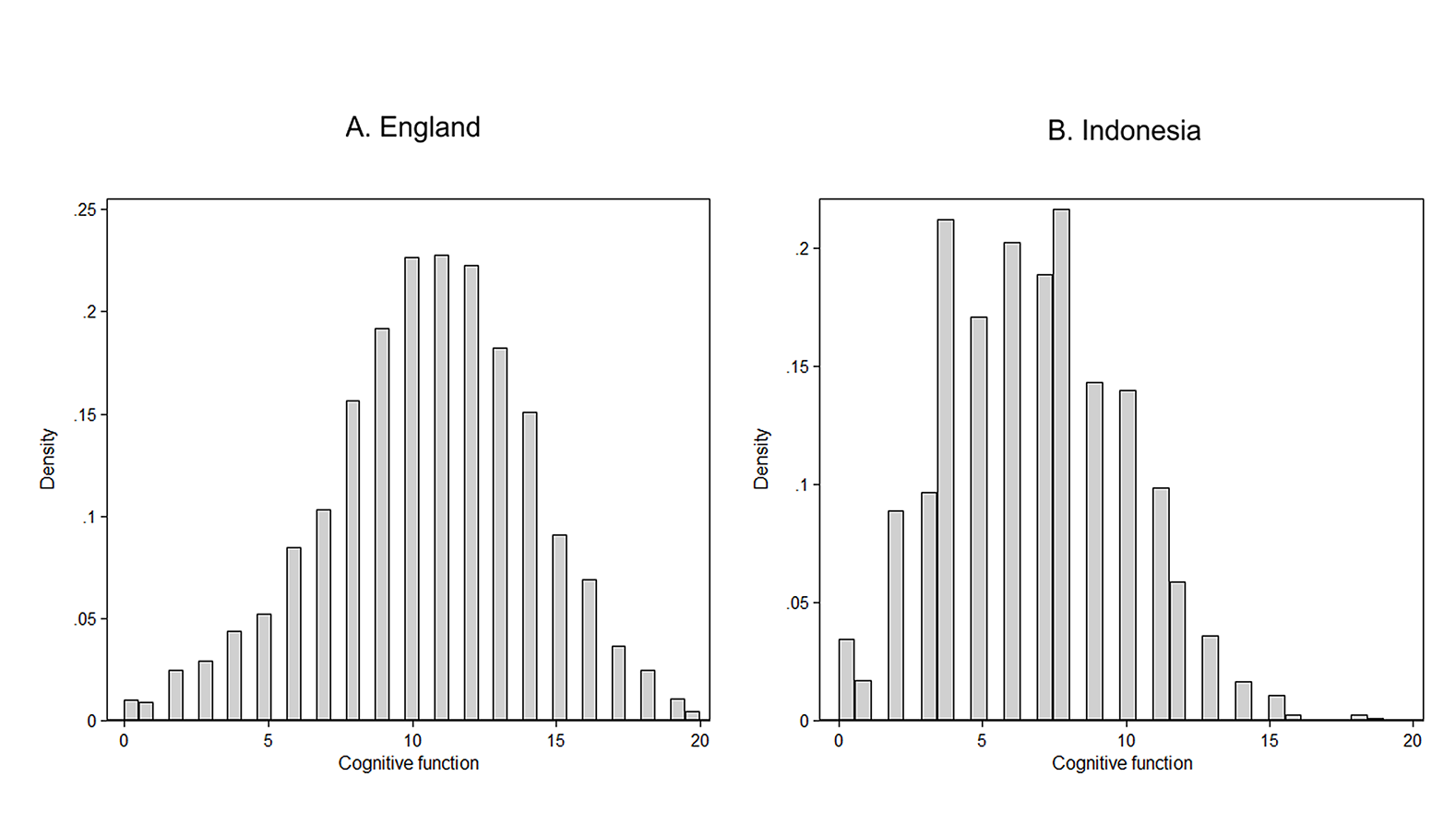

Supplement: S1 Fig — (TIF) [file pone.0148406.s003.tif]
